# Pus Formation

**Published:** 1887-09

**Authors:** 


### PUS FORMATION.

The paper by Dr. Black, in this number, although long, will well repay careful study. Let no practicing dentist imagine that it is too "scientific" for his comprehension, for he will find it as practical as a potato, and quite as full of nutriment. He may not be a believer in the bacterian origin of disease, but as an intelligent man he should be able to comprehend its theories, and to this end the paper will lend great assistance. He may be quite ignorant of the whole subject, and in this case he should read it a number of times. Those who desire to know anything concerning the septic conditions which it is so large a portion of a dentist's duties to combat, will give the paper earnest study, for here is one consistent explanation of the phenomena presented by pus under varying circumstances.

